# Long-lived lunar volcanism sustained by precession-driven core-mantle friction

**DOI:** 10.1093/nsr/nwad276

**Published:** 2023-10-31

**Authors:** Shuoran Yu, Xiao Xiao, Shengxia Gong, Nicola Tosi, Jun Huang, Doris Breuer, Long Xiao, Dongdong Ni

**Affiliations:** State Key Laboratory of Lunar and Planetary Sciences, Macau University of Science and Technology, Macau 999078, China; Planetary Science Institute, State Key Laboratory of Geological Processes and Mineral Resources, School of Earth Sciences, China University of Geosciences, Wuhan 430074, China; CAS Key Laboratory of Planetary Sciences, Shanghai Astronomical Observatory, Shanghai 200030, China; Institute of Planetary Research, German Aerospace Centre (DLR), Berlin 12489, Germany; Planetary Science Institute, State Key Laboratory of Geological Processes and Mineral Resources, School of Earth Sciences, China University of Geosciences, Wuhan 430074, China; Institute of Planetary Research, German Aerospace Centre (DLR), Berlin 12489, Germany; Planetary Science Institute, State Key Laboratory of Geological Processes and Mineral Resources, School of Earth Sciences, China University of Geosciences, Wuhan 430074, China; State Key Laboratory of Lunar and Planetary Sciences, Macau University of Science and Technology, Macau 999078, China

**Keywords:** Moon, precession, orbit, volcanism

## Abstract

Core-mantle friction induced by the precession of the Moon’s spin axis is a strong heat source in the deep lunar mantle during the early phase of a satellite’s evolution, but its influence on the long-term thermal evolution still remains poorly explored. Using a one-dimensional thermal evolution model, we show that core-mantle friction can sustain global-scale partial melting in the upper lunar mantle until ∼3.1 Ga, thus accounting for the intense volcanic activity on the Moon before ∼3.0 Ga. Besides, core-mantle friction tends to suppress the secular cooling of the lunar core and is unlikely to be an energy source for the long-lived lunar core dynamo. Our model also favours the transition of the Cassini state before the end of the lunar magma ocean phase (∼4.2 Ga), which implies a decreasing lunar obliquity over time after the solidification of the lunar magma ocean. Such a trend of lunar obliquity evolution may allow volcanically released water to be buried in the lunar regolith of the polar regions. As a consequence, local water ice could be more abundant than previously thought when considering only its accumulation caused by solar wind and comet spreading.

## Teaser

Precession-driven core-mantle friction can maintain a long-lived volcanism on the Moon until ∼3.1 Ga. Modelling suggests the Cassini state transition before the end of lunar magma ocean phase (∼4.2 Ga), which allows a decreasing lunar obliquity over time and the deposition of water ice in the lunar polar regions afterwards.

## INTRODUCTION

Although the Moon is a small rocky satellite, it still presents an anomalously long volcanic history spanning over ∼3.9–1.2 Ga [1], during which the emplacement of mare basalts occurred mostly on the lunar nearside. Such a long-lived volcanic activity raises a classic problem regarding the energy sources driving the thermal evolution of the Moon. Traditionally, the thermal evolution of the Moon is considered to be largely driven by the heat released by the decay of long-lived radioactive elements (Th, U and K). After Clementine and Lunar Prospector missions, it was realised that radioactive elements are highly enriched on the nearside, in the crust underlying the Procellarum KREEP Terrane (PKT) [2–5]. Indeed, radiogenic heating from the PKT can maintain the mantle below partially molten over most of the lunar history [6,7]. In addition, the early lunar mantle may have experienced a compositional overturn driven by the sinking of dense ilmenite-bearing cumulates (IBCs), a cumulate layer that formed when ∼90 vol. % of the lunar magma ocean (LMO) solidified [8–11]. Also being enriched in radioactive elements, the foundered IBC can subsequently heat up and buoyantly rise up via solid-state convection in the form of a degree-1 mantle upwelling [12,13]. The accompanying decompression melting provides an alternative explanation for the volcanic activity on the lunar nearside.

Nevertheless, this traditional view may need to be complemented by accounting for the effects of core-mantle friction induced by the precession of the lunar axis detected by the Apollo lunar laser ranging (LLR) experiment [14]. Although the dissipated power associated with core-mantle friction is just ∼6 × 10^7^ W at present, it was much more significant during the early phases of lunar evolution owing to a higher obliquity and more rapid rotation at the much shorter Earth-Moon distance [15]. Therefore, core-mantle friction is another important heat source in the lunar mantle, whose influence on the thermal evolution is yet to be examined.

## ORBITAL AND INTERIOR EVOLUTION MODEL

During the evolution, the semi-major axis of the lunar orbit (*a*) tends to increase towards the present-day value of ∼60.2 *R_E_* (*R_E_* is the Earth’s radius), which in turn continuously alters the rotational state of the Moon. Core-mantle friction begins when *a* ≈ 26–29 *R_E_* owing to the misalignment between the rotation axes of the mantle and core [16,17]. Since then, this friction dissipates rotational kinetic energy, thereby heating up the core-mantle boundary (CMB) with a power that scales as [14]


(1) \begin{eqnarray*}
P_f \propto \kappa \rho r^5 \sin ^3{I_e} a^{-9/2},
\end{eqnarray*}

where κ is a dimensionless parameter depending on the kinematic viscosity of the core, ρ is the density of the core, *r* is the radius of the frictional boundary (i.e. the core-mantle boundary), *I_e_* is the equatorial inclination angle (i.e. the angle between the spin axis and the normal to the ecliptic) and *a* is the semi-major axis of the lunar orbit. For a detailed discussion of the derivation of Equation (1), we refer the reader to the online supplementary material. Here we assume the kinematic viscosity and density of the lunar core to be constant in time. Correspondingly, parameter κ also remains constant. Based on Equation (1), the power of core-mantle friction can be estimated as


(2) \begin{eqnarray*}
P_f = P_{f,0} \bigg ( \frac{\sin {I_e( a)}}{\sin {I_{e,0}}} \bigg )^3 \bigg ( \frac{a}{a_0} \bigg )^{-9/2},
\end{eqnarray*}

where *P*_*f*,0_, *I*_*e*,0_ and *a*_0_ are respectively the core-mantle friction power (∼6 × 10^7^ W), equatorial inclination (∼1.56^○^) and semi-major axis (60.2 *R_E_*) at present day. Note that the equatorial inclination angle is a function of the semi-major axis because the Moon is in the Cassini state [15,18].

**Figure 1.**
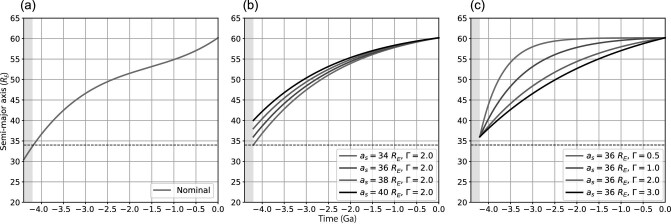
Time evolution of the semi-major axis for (a) the nominal lunar orbit according to [15], and for parameterised orbits with (b) *a_s_* = 34–40 *R_E_* and Γ = 2.0 and (c) *a_s_* = 36 *R_E_* and Γ = 0.5–3.0, where *R_E_* is the Earth’s radius. The dashed black line marks the semi-major axis for the transition to the current Cassini state. The grey box specifies the lunar magma ocean (LMO) phase ending at ∼4.2 Ga.

The evolution of the lunar orbit, particularly its early phase, is not well constrained. As an example, Fig. 1(a) shows the time evolution of the semi-major axis for a nominal lunar orbit model [15]. In order to test the influence of different orbital evolutions, we parameterise the semi-major axis by an exponential function of time increasing from *a_s_* at the end of the LMO phase towards the present-day value of ∼60.2 *R_E_*, where a factor Γ is used to control the rate of semi-major axis increase (see Fig. 1(b) and 1(c) and the online supplementary material).

Using a one-dimensional (1D) parameterised stagnant-lid mantle convection model (see the online supplementary material), we simulate the thermal evolution of the Moon from the end of the LMO phase ∼4.2 Ga [19] until the present day. We consider a wet Moon with a bulk water content of 100 ppm, favoured by the analysis on the lunar picritic glasses [20–22], and a much drier Moon with a bulk water content of only 1 ppm. The reference viscosity (η_*r*_), i.e. the viscosity of the water-free mantle calculated at 1600 K, is considered as a free parameter. In accordance with the solidification of the LMO, the initial mantle temperature is obtained by averaging volumetrically the solidus temperature for all depths in the lunar mantle (see the online supplementary material). In order to account for the effect of strong tidal heating in the early lunar crust [23] and for the possibility that heating due to core-mantle friction started during the LMO phase, we also allow the initial mantle temperature to increase by an amount Δ*T_m_*, which is also used as a free parameter.

The CMB temperature is evolved through the energy conservation equation for the lunar core, including the heat due to core-mantle friction (see the online supplementary material). We further assume that the core is initially superheated with respect to the mantle by 300 K. Note however that this value is just used to initiate the simulations and to ensure numerical stability in the calculation of the bottom thermal boundary layer. After a short adjustment phase, the CMB temperature is quickly controlled by core-mantle friction and is therefore not sensitive to the initial choice.

Earlier work also suggested that core-mantle friction would remelt the deep lunar mantle, which in turn would weaken the core-mantle friction itself [15]. We note however that this molten layer cannot likely be stable at the CMB owing to the upward percolation of the melt (see the online supplementary material). Therefore, no melt is retained in the deep mantle. Accordingly, core-mantle friction can take place continuously. To account for the effect of upward melt percolation from the CMB, the CMB temperature is not increased further after reaching the local solidus. In addition, we consider a percolation heat flux in the energy conservation equations for the lunar mantle, which is zero if the CMB temperature is sub-solidus and becomes positive as soon as the CMB temperature reaches the local solidus (see the online supplementary material).

Sinking of the IBC near the end of the LMO phase leads to the enrichment of iron and titanium in the deep mantle [8–11]. The foundered IBC could melt owing to the high abundance of radioactive elements [24] and the high power of the core-mantle friction. Yet, the generated melt may not percolate upwards because of the high density associated with the high iron and titanium content. Correspondingly, the melt of the foundered IBC might form a dense basal magma ocean over the CMB, which may weaken the core-mantle friction [15]. However, even if the basal magma ocean weakens the friction at the CMB, it allows the other solid-liquid friction to occur at its top. Besides, the thermal and mechanical properties of a basal magma ocean resemble those of the core [25,26]. Thus, our model is still applicable to the mantle above the basal magma ocean. Even so, it is challenging to realistically evaluate melt-mantle friction at the interface between the solid mantle and magma because the data of the LLR experiment were analysed by assuming a simple core-mantle stratification in the deep lunar interior. As a trade-off option, this work just focuses on the simplest core-mantle friction.

In the online supplementary material we perform a parameter study to examine the influence of core-mantle friction on the thermal evolution of the Moon. Based on the results, core-mantle friction can significantly affect the thermal evolution of the Moon, in particular by promoting partial melting of the upper mantle. In addition, the thermal evolution of the Moon strongly depends on the evolution of the lunar orbit as controlled by parameters *a_s_* and Γ introduced above. In order to determine possible evolution scenarios of the semi-major axis, we consider three main constraints on the thermal state of the lunar interior. First, the upper mantle temperature at the end of the simulations must agree with the present-day temperature profile, which is constrained by the Apollo seismic experiment [27] and electrical conductivity data [28,29]. Second, to account for the transition of viscous relaxation for pre-Nectarian impact basins [30], the temperature at the crust-mantle interface must drop below ∼1300 K before 4.45–4.21 Ga [31]. Third, lunar picritic glasses suggest that the depth of multiple saturation does not exceed ∼540 km [32–34], which poses a constraint on the maximum depth of lunar mantle melting.

## RESULTS

**Figure 2.**
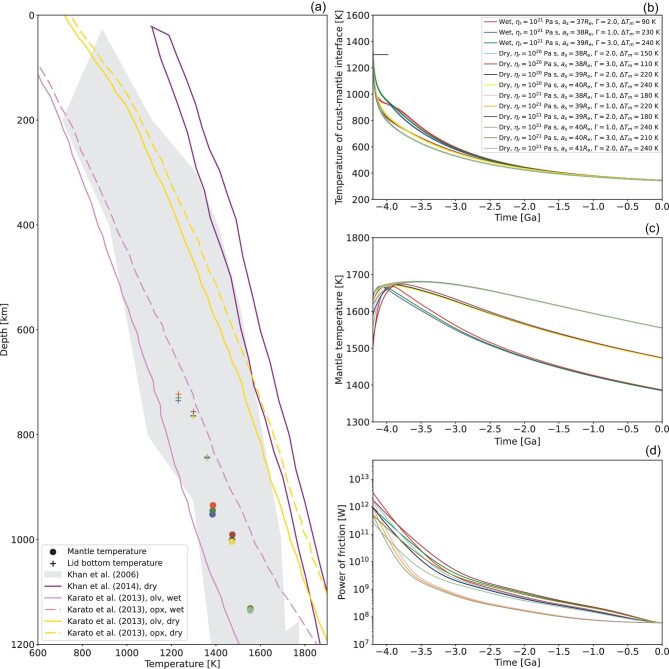
(a) Comparison between the present-day temperature at the bottom of the stagnant lid (plus symbols) and upper mantle temperature (circles) of all successful cases. The gray area indicates the range of possible temperature profiles of the lunar mantle over a depth of 0–1200 km inferred from Apollo seismic data [27]. Purple curves specify the range of the lunar mantle temperature inferred from the electrical conductivity data by assuming a dry mantle with its typical composition [29]. Pink and gold curves denote mantle temperatures inferred from electrical conductivity data by assuming a wet mantle with a bulk water content of 100 ppm and a dry mantle, respectively [28], with solid and dashed lines referring to an olivine (solid) and orthopyroxene (dashed) composition, respectively [28]. (b) Time evolution of the temperature at the crust-mantle boundary for the successful cases. The black bar indicates the critical temperature of 1300 K corresponding to the transition in relaxation of pre-Nectarian basins [30]. (c) Time evolution of the mantle temperature and (d) of the frictional power for the successful cases.

All successful cases are summarised in the online supplementary material, with the corresponding thermal evolution plotted in Fig. 2. As shown in Fig. 2(a), the modelled present-day mantle temperature and lid bottom temperature are in good agreement with the temperature profile of the lunar mantle inferred from Apollo seismic data [27]. Nevertheless, we note that the estimation based on Apollo seismic experiments did not consider the possibility of a wet lunar mantle. For a better comparison, we also use the lunar mantle temperature inferred from the data of electrical conductivity by taking into account the composition and water content of the lunar mantle itself [28,29]. The present-day lunar mantle temperatures in the wet-Moon cases agree better with the experimental values inferred with a bulk-mantle water content of 100 ppm, whereas the present-day mantle temperatures in the dry-Moon cases are systematically lower than those inferred for a dry lunar mantle.

**Figure 3.**
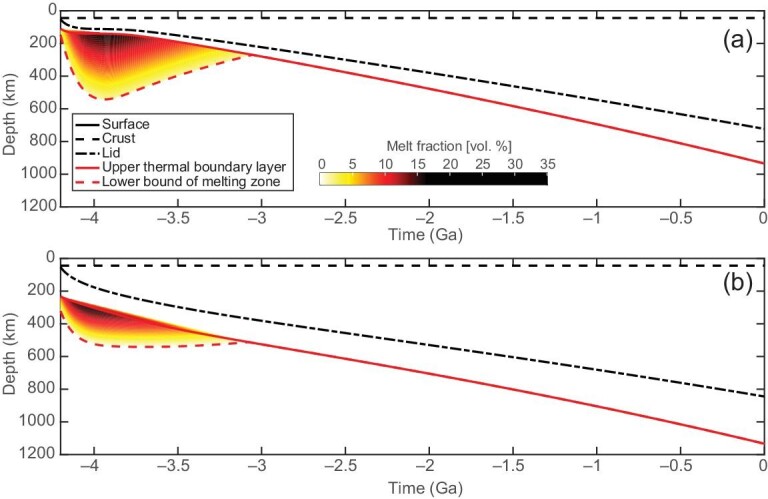
Time evolution of the stagnant lid thickness (dash–dot lines) and partial melt region (contour) for (a) a wet-Moon case with η_*r*_ = 10^21^ Pa s, *a_s_* = 37 *R_E_*, Γ = 2.0 and Δ*T_m_* = 90 K, and (b) a dry-Moon case with η_*r*_ = 10^21^ Pa s, *a_s_* = 38 *R_E_*, Γ = 1.0 and Δ*T_m_* = 180 K. The black dashed line indicates the thickness of the primordial crust, which is held fixed throughout the simulations. The solid and dashed red lines indicate the depths of the upper thermal boundary layer and the bottom of the partial melt region, respectively. The coloured contour represents the volumetric melt fraction in the partially molten mantle. In panel (a), the maximum depth of the melt region is 542.70 km and is attained at 3.94 Ga, with mantle melting ending at 3.06 Ga. In panel (b) the maximum depth of the melt region is 541.80 km and is attained at 3.70 Ga, with mantle melting ending at 3.08 Ga

Figure 2(b) shows the evolution of the temperature at the crust-mantle interface, which, for all successful cases, is always lower than the critical value of ∼1300 K after 4.2 Ga, consistent with the transition of the relaxation mode for pre-Nectarian basins before the end of the LMO phase [30,31]. Figure 2(c) and 2(d) show the evolution of the mantle temperature and frictional power for all successful cases, which can be adopted to evaluate the evolution of mantle melting. Note that the mantle temperature here is sampled at the bottom of the upper boundary layer. Figure 3 shows the evolution of partial melting for a specific successful simulation. Results corresponding to other cases are reported in the online supplementary material. Our model suggests that core-mantle friction can sustain long-lived partial melting in the upper mantle until 3.18–2.88 Ga. During this time, the maximum depth of partial melting, indicative of a peak in the lunar volcanic activity, occurs between 4.09 and 3.61 Ga.

**Figure 4.**
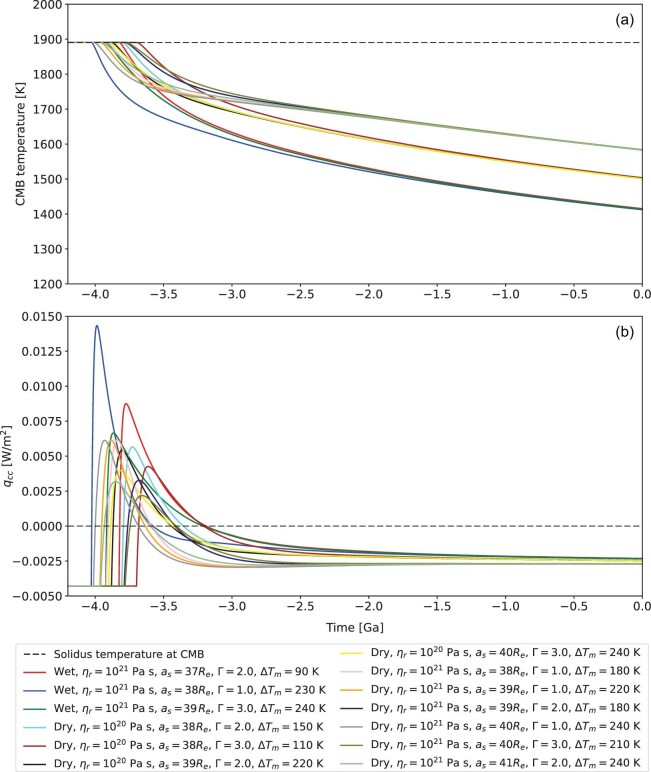
(a) Time evolution of the CMB temperature for all successful cases. The dashed line notes the solidus temperature of the lunar mantle at the CMB. The cut-off of the CMB temperature relates to the percolation of the melt when the core-mantle friction is vigorous enough to remelt the bottom of the lunar mantle. (b) Time evolution of the heat flux *q_cc_* for all successful cases. The positive *q_cc_* indicates the activation of a core dynamo.

Figure 4(a) shows the time evolution of the CMB temperature for all successful cases. In the early phase of lunar evolution, core-mantle friction is vigorous enough to remelt the deep lunar mantle. Correspondingly, the CMB temperature is capped at the local solidus under the assumption that the generated melt will percolate upward. Actual cooling of the core begins at 4.03–3.70 Ga, when the frictional power sufficiently declines, and continues until the present day. Core cooling may also support core convection, which in turn allows the lunar dynamo to operate. In order to evaluate whether or not a core dynamo is possible, we define a heat flux


(3) \begin{eqnarray*}
q_{cc} = \frac{M_c c_{pc}}{4 \pi r_c^2} \, \frac{dT_c}{dt} - K_c \frac{\alpha _c g_c, T_c}{c_{pc}},
\end{eqnarray*}

where *M_c_* is the mass of the lunar core, *c_pc_* is the heat capacity, *K_c_* is the thermal conductivity (∼25 W/(m K)), *T_c_* is the CMB temperature and *g_c_* is the gravitational acceleration at the CMB. A positive *q_cc_* is indicative of a core dynamo. Figure 4(b) shows the time evolution of *q_cc_* for all successful cases. The value of *q_cc_* is initially negative. It becomes positive between 4.03 and 3.68 Ga, and negative again after 3.69–3.19 Ga. Correspondingly, in this scenario a lunar dynamo would only be active for a few hundred million years.

## DISCUSSIONS

In the previous works, the lunar thermal evolution is believed to be dominated only by the radioactive elements Th, U and K in the bulk Moon [7,12,35]. Our model suggests that precession-driven core-mantle friction can also affect the thermal evolution of the Moon and the melting of the lunar mantle. According to the constraints on the thermal conditions of the lunar interior based on the Apollo seismic experiments, relaxation of pre-Nectarian basins and lunar picritic glasses, our models suggest that *a_s_* is 37–41 *R_E_* and Δ*T_m_* is 90–240 K. Correspondingly, core-mantle friction can maintain partial melting in the upper lunar mantle until 3.18–2.88 Ga. Nevertheless, the estimation based on Apollo seismic experiments did not account for the possibility of the wet lunar mantle. For this reason, we also use the present-day lunar mantle temperature inferred from the data of electrical conductivity as the other constraint. In this case, our model favours a wet Moon with a bulk water content of 100 ppm, *a_s_* = 37–39 *R_E_* and Δ*T_m_* = 90–240 K. Correspondingly, the melting in the lunar mantle can be sustained until 3.12–3.06 Ga.

Our 1D model can only predict the laterally averaged thermal evolution of the lunar mantle. As core-mantle friction occurs over the whole CMB, the predicted melting of the upper mantle is likely to be a global phenomenon. In a more realistic Moon, the enrichment of radioactive elements within or beneath the crust underlying the PKT region can lead to an asymmetric thermal evolution of the Moon [6,7]. Nevertheless, it is unclear whether or not the lunar picritic glasses associated with the PKT are only representative of mantle melting occurring beneath the PKT itself [36]. Our simulations featuring a 10-fold increase of the Th abundance in the crust indicate that a strong crustal enrichment in Th does not significantly affect the maximum depth of melting and its onset time, but rather tends to extend the time over which partial melting can occur (see the online supplementary material). We argue that mantle melting responsible for the generation of the picritic glasses may have been a global phenomenon sustained by core-mantle friction, rather than the consequence of the enrichment of radioactive elements within or beneath the PKT.

According to the crater-counting chronology of mare basalts, the lunar volcanic activity started at ∼3.9 Ga or earlier when considering the lunar cryptomares [37,38], reached its maximum between 3.7 and 3.3 Ga, and then faded after ∼3.0 Ga [1,39]. This trend is roughly consistent with the evolution of upper mantle melting predicted by our models. In addition, lunar volcanism, with the exception of the activity within Mare Tranquilitatis, is generally characterised by a high TiO_2_ content (∼4 wt %) [39]. Accounting for the sinking of the IBC in the early lunar mantle [8–11], these volcanic activities are likely related to the melting of the upper lunar mantle. Hence, the melting of the upper lunar mantle maintained by core-mantle friction may account for the intense volcanic activities before ∼3.0 Ga.

A subsequent volcanic phase characterised by high-Ti content occurred around the centre of the PKT between ∼2.3 and 1.2 Ga [39]. Accounting for the high-Ti content and its spatial distribution, this phase may have been caused by local uprising of the foundered IBC. Furthermore, geodynamic models suggest that a mantle upwelling driven by shallow enrichment in heat-producing elements may have fueled long-lived volcanism in the PKT region [7,40]. This late volcanic phase was likely related to regional decompression melting associated with upwelling mantle flow, which does not necessarily require partial melting of the upper mantle at a global scale, as predicted by our model.

The crater counting chronology also suggests an early high-Ti basalt volcanism during 3.85–3.55 Ga [1]. Such an early Ti-rich volcanic phase occurred within and out of PKT and presents a TiO_2_ content of ∼4–8 wt %, much lower than that of the late high-Ti volcanic phase (8–12 wt %) [39]. In view of these two features, this early high-Ti basalt volcanism may relate to the Ti-rich sources at the shallow lunar mantle, associated with the IBC entrapped by the stagnant lid or the IBC-mantle mixture produced during the early lunar mantle overturn [39]. Both mechanisms were also manifested via geodynamic modelling [10]. If the upper lunar mantle was remelted during 3.85–3.55 Ga, these shallow Ti-rich sources would contribute the Ti-rich magma needed for the early high-Ti volcanism.

The presence of melt in the lunar mantle also tends to weaken its rheology, which may further influence the overall thermal evolution of the Moon. However, the melt-fraction dependence of the viscosity—which is a highly localised feature—can hardly be taken into account in the frame of 1D parameterised convection models such as those employed here, which are based on boundary layer theory and designed to treat global-scale heat transfer in a single-phase fluid [41,42]. As a first-order approximation, we tested the influence of the melt on the lunar thermal evolution by accounting for the volume-averaged melt fraction of the whole convective mantle in the reference viscosity (see the online supplementary material). Accounting for the melt-induced viscosity reduction causes the mantle to convect more vigorously and to cool more quickly, making it difficult to simultaneously satisfy all constraints described above. Nevertheless, several dry-Moon cases with a reference viscosity of 10^21^ Pa s do reproduce the constraints associated with the relaxation of pre-Nectarian and Nectarian basins, as well as the maximum melting depth suggested by the lunar picritic glasses. But these cases also yield present-day mantle temperatures that are generally lower than those inferred from electrical conductivity data under the assumption of a dry mantle. In addition, mantle melting can be sustained until ∼3.2 Ga, which suggests that precession-driven core-mantle friction plays an important role in maintaining long-lived lunar volcanism.

In our thermal evolution model, we consider a heat flux associated with melt percolation from the bottom of the lunar mantle in order to ensure the conservation of frictional heat. This percolative heat flux is important for maintaining long-lived mantle melting. For comparison, we also run models of thermal evolution neglecting the influence of melt percolation (see the online supplementary material). In this case, sustaining long-lived mantle melting until ∼3.0 Ga is still possible, which emphasises the crucial role played by frictional heating. However, the parameter space for which this condition can be met is much more restricted. It is limited to cases with a dry lunar mantle with a high reference viscosity of 10^21^ Pa s and high initial mantle superheat of Δ*T_m_* = 240 K.

Most volcanic activity on the Moon occurred on the nearside. Early works explain the eruption of magma by its positive buoyancy with respect to the lunar crust [43]. Accordingly, removal of the light feldspathic upper crust can promote the eruption of magma. Nevertheless, the lack of vigorous volcanic activity within the South Pole–Aitken (SPA) basin, where the light feldspathic upper crust was mostly removed [44,45], seems to be incompatible with upper mantle melting on a global scale. This discrepancy can be reconciled by considering the possibility of a hemispherical (i.e. degree-1) pattern of lunar mantle convection or the global asymmetry of iron content. In some works, it has been proposed that the vigorous volcanic activity on the lunar nearside is a consequence of a degree-1 pattern of mantle convection [12,13]. If a degree-1 upwelling flow formed below the lunar nearside, a corresponding downwelling flow should have formed beneath the farside, thus limiting magma formation and extrusion. Alternatively, hyperspectral remote-sensing observations indicate that the Mg$^\#$ of the farside is higher than that of the nearside crust by ∼10 on average [46]. According to this finding, the farside crust may originate from a relatively iron-depleted magma ocean, which would produce iron-depleted mantle cumulates upon crystallisation. The solidus temperature of iron-depleted peridotite is higher than that of iron-rich peridotite at the same pressure conditions [47]. Hence, mantle melting on the farside would be more difficult, which in turn would result in a relatively weak volcanic activity within the SPA.

Because of the high power during the early phase of lunar evolution, core-mantle friction has also been suggested as a possible energy source to power the lunar core dynamo until ∼3.0 Ga [15]. Nevertheless, this hypothesis was proposed by mapping the power of core-mantle friction directly into the power needed for maintaining a dipole magnetic field via the power-based scaling law, but was never examined in the context of lunar thermal evolution. According to our modelling results, core-mantle friction likely suppresses the operation of the core dynamo until 4.03–3.70 Ga and therefore cannot explain the whole history of the lunar magnetic field, which has likely been present as recently as until ∼1.0 Ga [48,49]. Thus, other mechanisms such as precession-driven friction at the inner core boundary [50] or the release of latent heat that accompanies the growth of an inner core growth [40,51] may be required.

In our model, we neglect the influence of tidal dissipation on the lunar thermal evolution. In the online supplementary material, we present a comparison for the power associated with radiogenic heating, core-mantle friction and tidal dissipation. The latter is significantly lower than the power of core-mantle friction if the semi-major axis is less than ∼46 *R_E_*. When the power of tidal dissipation becomes higher than the power of core-mantle friction, radiogenic heating dominates the thermal evolution of the Moon. Therefore, tidal dissipation is always a secondary heat source for the lunar thermal evolution in comparison with radiogenic heating and core-mantle friction (the two heat sources considered in our model), and can thus be neglected.

In this work, we simply used an exponential function to describe the time evolution of the semi-major axis. Nevertheless, our model suggests that lunar volcanic activity can be used as an indicator to potentially constrain the evolution of the lunar orbit. In particular, a well-constrained lunar orbit is critical for evaluating time variations of the lunar spin axis, which influences the deposition of water ice and the formation of permanently shaded regions (PSRs). The deposition of water ice cannot occur near the Cassini state transition owing to the high lunar obliquity, but becomes possible afterwards because of the decreasing lunar obliquity over time [52]. As our models constrain *a_s_* to be 37–38 *R_E_*, the transition of the Cassini state should occur before the end of the LMO phase. Correspondingly, the deposition of water ice could take place over most of the lunar history. In addition, lunar volcanic activity from ∼3.9 until 1.2 Ga may lead to the release of mantle volatiles and the formation of a transient atmosphere [53]. If the bulk Moon was water rich as the lunar picritic glasses indicate [20–22], such a transient atmosphere may allow a considerable fraction of water to be buried in the lunar regolith of the polar regions, which was in turn entrapped until the lunar obliquity was small enough to form PSRs. Correspondingly, the water ice in PSRs would be more abundant than what we thought previously when considering only the injection of solar wind and comets. This speculation can be verified by the coming sampling missions for the lunar polar regions.

## CONCLUSIONS

We examined the influence of precession-driven core-mantle friction on the lunar thermal evolution. Using various constraints on the thermal state of the lunar interior, our model suggests a wet Moon with a bulk water content of 100 ppm, a semi-major axis of 37–39 *R_E_* by the end of the LMO phase and a significantly superheated initial lunar mantle. Correspondingly, core-mantle friction can sustain partial melting in the upper lunar mantle until 3.12–3.06 Ga. Such a long-lived upper mantle melting may account for the intense volcanic activities before ∼3.0 Ga inferred from crater-counting chronology. The enrichment of radioactive elements in the lunar crust does not affect the mantle melting before ∼3.0 Ga, but can prolong the duration of mantle melting. As a consequence, a high crustal heat production may be important only for maintaining or enhancing the late volcanic activities. The degree-1 pattern of lunar mantle convection or the global asymmetry of iron content should be invoked to explain the focusing of volcanism on the lunar nearside and the lack thereof within the SPA. In addition, the inferred *a_s_* favours the Cassini state transition before the end of the LMO phase, i.e. ∼4.2 Ga, which implies a decreasing lunar obliquity over time during most of lunar history. Water degassed upon long-lived volcanic activity might be buried in the lunar regolith of polar regions. Therefore, local water ice could be more abundant than what was previously thought when considering only its injection via solar wind and comets. This speculation can be verified by upcoming sampling missions for the lunar polar regions.

## Supplementary Material

nwad276_Supplemental_Files
