# Supplementary material for: Long-lived lunar volcanism sustained by precession-driven core-mantle friction
Source: Natl Sci Rev. 2023 Oct 31;11(2):nwad276. doi: 10.1093/nsr/nwad276 (PMC10776352; doi:10.1093/nsr/nwad276)
Supplement: nwad276_Supplemental_Files [file nwad276_supplemental_files.zip › si_final.pdf]

Supplementary Information for "Long-lived lunar volcanism sustained by precession-driven core-mantle friction"

Shuoran Yu[1,*,†], Xiao Xiao[2,†], Shengxia Gong[3],
Nicola Tosi[4], Jun Huang[2], Doris Breuer[4], Long Xiao[2], Dongdong Ni[1]

[1] State Key Laboratory of Lunar and Planetary Sciences, Macau University of Science and Technology, Macau SAR 999078, China.
[2] Planetary Science Institute, State Key Laboratory of Geological Processes and Mineral Resources, School of Earth Sciences, China University of Geosciences, Wuhan 430074, China.
[3] CAS Key Laboratory of Planetary Sciences, Shanghai Astronomical Observatory, Shanghai 200030, China.
[4] Institute of Planetary Research, German Aerospace Centre, Berlin 12489, Germany.

[*] Corresponding author. E-mail: shuoran.yu@icloud.com
[†] Equal contributions to this work.

Supplementary Text 1. Thermal evolution model

We simulate the global thermal evolution of the lunar interior using a 1-D parameterised stagnant-lid mantle convection model in spherical geometry including the influence of mantle melting (e.g., [1]). Figure S1 shows the thermal structure in the convective lunar mantle. In contrast to previous settings, here we account for the fact that core-mantle friction may remelt the deep lunar mantle if the frictional heat cannot be dissipated eventually by mantle convection. As a consequence, the generated melt can percolate upwards and in turn allow an extra heat transfer in addition to the heat transfer via mantle convection (Supplementary Text 6). By considering this effect, the thermal evolution of the lunar mantle is modeled by an energy conservation equation:

$$(1 + St)\,\rho_m c_{pm} V_m \epsilon_m \frac{dT_m}{dt} = -q_{mu} A_{mu} + q_{mb} A_c + P_{perc} + \rho_m Q_m V_m \tag{1}$$

where St is the Stefan number, ρ_m is the mantle density, c_{pm} is the mantle heat capacity, V_m is the mantle volume, ϵ_m is the ratio between volume-averaged mantle temperature and upper mantle temperature, T_m is the upper mantle temperature, t is the time, q_{mu} is the heat flux over the upper boundary layer, A_{mu} is the area of the upper boundary layer, q_{mb} is the heat flux over the lower boundary layer, P_{perc} is the power budget due to upward melt percolation from the core-mantle boundary (CMB), A_c is the area of the CMB, and Q_m is the volumetrically-averaged heat production rate of the mantle. The Stefan number controls the release of latent heat and is given by

$$St = \frac{L}{c_{pm} V_m} \int_{V_{melt}} \frac{d\phi}{dT} dV \tag{2}$$

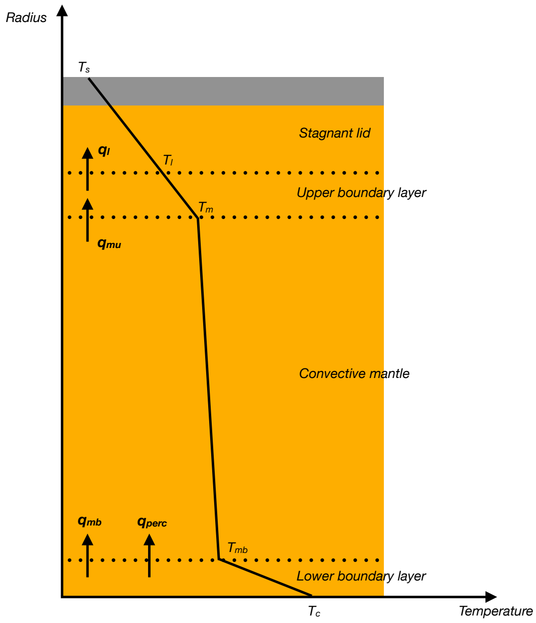

Figure S1: Thermal structure in the convective lunar mantle. The grey layer on top represents the lunar primary crust, which is fixed in the model.

where L is the specific latent heat of melting, ϕ is the volumetric fraction of melt, V_{melt} is the volume of the molten zone. The lid bottom temperature is calculated in dependence of the upper mantle temperature as follows

$$T_l = T_m - \Theta \frac{RT_m^2}{E}, \tag{3}$$

where R is the gas constant, E is the mantle activation energy and Θ is a dimensionless coefficient [2]. Here, to account for spherical geometry, we use $\Theta = 2.9$ [3].

The heat fluxes through the two boundary layers are calculated on the base of boundary layer theory [4]. The thickness of the upper boundary layer is given by

$$\delta_{mu} = (r_l - r_c) \left(\frac{Ra_{mu}}{Ra_{cr}} \right)^{-1/3}, \tag{4}$$

where r_l is the radius of the lid bottom, r_c is the core radius, Ra_{mu} is the local Rayleigh number, Ra_{cr} is the critical Rayleigh number, i.e. the minimum Rayleigh number allowing convection to take place. The local Rayleigh number is given by

$$Ra_{mu} = \frac{\alpha_m \rho_m^2 c_{pm} g_{mu} \Delta T \, (r_l - r_c)^3}{K_m \eta_{mu}}, \tag{5}$$

where α_m is the thermal expansivity of the mantle, g_{mu} is the gravitational acceleration at the upper boundary layer estimated by Eq. (6.12) in [4], ΔT is the sum of the temperature differences across two boundary layers, r_l is the radius of the lid bottom, r_c is the core radius, K_m is the thermal conductivity of the mantle, and η_{mu} is the viscosity at the upper boundary layer. The

heat flux through the upper boundary layer can then be expressed as

$$q_{mu} = K_m \frac{T_m - T_l}{\delta_{mu}}. \tag{6}$$

The thickness of the lower boundary layer is calculated as follows [5]

$$\delta_{mb} = \left(\frac{K_m \eta_c Ra_{i,cr}}{\alpha_m \rho_m^2 c_{pm} g_s \left(T_c - T_{mb}\right)} \right)^{1/3}, \tag{7}$$

where η_c is the viscosity estimated at the average temperature of the lower boundary layer, T_c is the CMB temperature, T_{mb} is the temperature at the top of lower boundary layer, $Ra_{i,cr}$ is the critical Rayleigh number at lower boundary layer. The temperature at the top of the lower boundary layer is obtained by imposing a (linearised) adiabatic temperature profile in the convecting part of the mantle and is given by

$$T_{mb} \approx T_m + \frac{\alpha_m g_s T_m}{c_{pm}} \left(r_l - r_c - \delta_{mu} - \delta_{mb}\right). \tag{8}$$

The local critical Rayleigh number $Ra_{i,cr}$ is determined by [5]

$$Ra_{i,cr} = 0.28 Ra_i^{0.21}. \tag{9}$$

where $Ra_i = \alpha_m \rho_m^2 c_{pm} g_s \Delta T^* \left(r_p - r_c\right)^3 / K_m \eta_{mu}$ with the planetary radius r_p and $\Delta T^* = (T_m - T_s) + (T_c - T_{mb})$. Similarly, the heat flux through the lower boundary layer can be determined as

$$q_{mb} = K_m \frac{T_c - T_{mb}}{\delta_{mb}}. \tag{10}$$

The mantle viscosity is calculated using the Arrhenius power-law for a wet mantle [6, 1], i.e.

$$\eta = \frac{\eta_r}{X_w^m} \exp\left(\frac{E + pV}{RT} - \frac{E + pV}{RT_r}, \right) \tag{11}$$

where η_r is the reference viscosity at the reference temperature T_r (1600 K), X_w^m is the average water content of the solid mantle (expressed in ppm), E is the activation energy, p is the hydrostatic pressure, V is the activation volume, and R is the gas constant. We note that Eq. (11) can result in a nearly infinite viscosity if the water content is low. To avoid this problem, we truncate the estimated viscosity of wet mantle by the viscosity of dry mantle for the same conditions, i.e. $\eta_{dry} = \eta_r \exp\left[(E + pV)/RT - (E + pV)/RT_r\right]$.

The stagnant lid is the cold and stiff layer on the top of the lunar interior that does not take part in the mantle convection. We use the steady-state heat conduction equation to model the temperature distribution in the stagnant lid, i.e.

$$\frac{1}{r^2} \frac{\partial}{\partial r} \left(r^2 K \frac{\partial T}{\partial r} \right) + Q = 0 \tag{12}$$

where Q is the heat production rate, K is the thermal conductivity. The boundary conditions for Eq. (12) are

$$T\left(r_p\right) = T_s, \tag{13}$$

$$T\left(r_l\right) = T_l, \tag{14}$$

where r_p is the planetary radius, r_l is the bottom lid radius, T_s and T_l are the surface and bottom lid temperatures, respectively. The evolution of the stagnant-lid thickness is determined by an energy balance at the bottom of the lid, i.e.

$$\rho_m c_{pc} (T_m - T_l) \frac{dD_l}{dt} = q_l - q_{mu} \tag{15}$$

where D_l is the thickness of the stagnant lid and q_l is the heat flux through the lid bottom.

The CMB temperature depends on the energy conservation of the lunar core. For simplicity, we consider a core-cooling condition without the release of latent heat that would accompany the crystallisation of an inner core. Assuming a convective core, its temperature profile is adiabatic, i.e.

$$\frac{dT_{core}}{dr} = -\frac{\alpha_c g_c T_{core}}{c_{pc}}. \tag{16}$$

where α_c is the thermal expansivity of the core, g_c is the gravitational acceleration in the core, and c_{pc} its heat capacity. Assuming a constant core density, the gravitational acceleration over the radius is given by

$$g_c(r) = \frac{4}{3}\pi G \rho_c r, \tag{17}$$

where G is the gravitational constant, and ρ_c the core density. By substituting Eq. (17) into Eq. (16), we obtain

$$\frac{dT_{core}}{dr} = -\frac{4\pi G \alpha_c \rho_c r}{3c_{pc}}, \tag{18}$$

which can be integrated to give

$$T_{core}(r) = T_c \exp\left(\frac{r_c^2 - r^2}{H_{c,ad}^2}\right), \tag{19}$$

where r_c is the core radius and $H_{c,ad} = \sqrt{3c_{pc}/2\pi G \alpha_c \rho_c}$ is the adiabatic scale height. For the lunar core, $H_{c,ad}$ is $\sim 10^8$ m and therefore is far greater than the core radius $\sim 10^5$ m. Thus, the lunar core can be reasonably assumed to be isothermal.

Accounting for core-mantle friction, the temperature of CMB is computed with the following energy conservation equation

$$\rho_c c_{pc} V_c \frac{dT_c}{dt} = \begin{cases} 0, \text{if } T_c = T_{sol,c} \text{ and } P_f > 4\pi r_c^2 q_{mb}, \\ -q_{mb} A_c + P_f, \text{ otherwise} \end{cases} \tag{20}$$

where ρ_c is the core density, c_{pc} is the core heat capacity, V_c is the volume of the core, $T_{sol,c}$ is the solidus temperature at the CMB, A_c is the area of the CMB, and P_f is the power of core-mantle friction. We note that the core-mantle friction can remelt the overlying solid mantle when the CMB temperature reaches the local solidus temperature and the frictional heat cannot be dissipated eventually via the heat conduction of lower boundary layer. In Supplementary Text 5, we show that the generated melt can percolate downwards eventually. Correspondingly, the CMB temperature will remain at the local solidus temperature and not increase further.

To treat mantle melting, we assume a linear relation to compute at each time step the melt fraction

$$\phi = \frac{T - T_{sol}}{T_{liq} - T_{sol}}, \tag{21}$$

where T_{sol} is the solidus temperature and T_{liq} is the liquidus temperature. Accounting for the influence of water, the solidus and liquidus temperature are given by [7]

$$T_{sol}\left(p, X_w^{melt}\right) = T_{sol,dry}\left(p\right) - k{X_w^{melt}}^{\gamma} \tag{22}$$

and

$$T_{liq}\left(p, X_w^{melt}\right) = T_{liq,dry}\left(p\right) - k{X_w^{melt}}^{\gamma} \tag{23}$$

where p is the pressure in GPa, X_w^{melt} is the water partitioned into melt, $T_{sol,dry}$ and $T_{liq,dry}$ are the solidus and liquidus temperature of the dry mantle, $k = 43$ K/wt.% and $\gamma = 0.75$. The water content in the melt is calculated as

$$X_w^{melt} = \frac{X_w^m}{\phi}\left[1 - \left(1-\phi\right)^{1/d_w}\right] \tag{24}$$

where d_w is the partition coefficient (~ 0.01) [7]. The water content in the melt is also limited by a pressure-dependent saturation point, i.e.

$$X_{w,sat}^{melt} = \chi_1 p^{\lambda} + \chi_2 p, \tag{25}$$

where $\chi_1 = 12.0$ wt. %/GPa$^{-\lambda}$, $\chi_2 = 1.0$ wt. %/GPa, $\lambda = 0.6$ [7]. For the dry mantle, we consider solidus and liquidus of dry KLB-1 peridotite [8], i.e.

$$T_{sol,dry}\left(p\right) = 1409 + 134.2p - 6.581p^2 + 0.1054p^3, \tag{26}$$

$$T_{liq,dry}\left(p\right) = 2035 + 57.46p - 3.4872p^2 + 0.0769p^3. \tag{27}$$

Supplementary Text 2. Power of core-mantle friction

The power dissipated at the CMB was previously derived in [9] and we present here the detailed mathematical derivation process. Since the core of the Moon was proved to be at least partially molten, dissipation at the CMB is expected when the fluid core moves at a different rate than the overlying solid mantle. By a simplified model based on the average fluid rotation, the power dissipated at the CMB can be determined as

$$P_f = -K(\vec{\omega} - \vec{\omega}')^2, \tag{28}$$

where $\vec{\omega}$ and $\vec{\omega}'$ are the angular velocities of mantle and core, respectively, K is a dissipation parameter defined as

$$K = \frac{3}{4}\pi^2\kappa\,\rho\,r^5|\vec{\omega}' - \vec{\omega}|. \tag{29}$$

Here ρ is the density of the liquid core, r is the core radius, and κ is a dimensionless parameter that depends on the viscosity of liquid core. The core-mantle spin rate difference can be evaluated by

$$|\vec{\omega}' - \vec{\omega}| = \frac{n \sin I_e}{\sqrt{1+\xi^2}}, \tag{30}$$

where n is the mean motion of the Moon, I_e is the Moon's equatorial inclination angle, and ξ is a coefficient that quantifies the core-mantle coupling. Note that the mean motion of the Moon n is proportional to $a^{-3/2}$ where a is the semi-major axis of the lunar orbit. For the strong core-mantle coupling, $\xi \gg 1$ and the spin axis of the core is lined up with that of the mantle.

For the weak core-mantle coupling, $\xi \ll 1$ and the spin axis of the core is nearly normal to the ecliptic plane. Here we set $\xi \ll 1$. By summarising all equations above, the power of core-mantle friction can be scaled as

$$P_f \propto \kappa\,\rho\,r^5 \sin^3 I_e\, a^{-9/2}. \tag{31}$$

Supplementary Text 3. Model parameters

Numerical values of the model parameters are reported in Table S1. According to GRAIL observations, the thickness of the lunar crust is set to 45 km [10]. In accordance with the results of Apollo Lunar Laser Ranging (LLR) experiment, we use a core radius of 352 km [9]. The thermophysical parameters of crust, mantle and core are set to typical values. The densities and Th abundances of the lunar crust and mantle are taken from the simulation of LMO crystallisation based on the software alphaMELTS as in [11]. In that model, we use the bulk composition in [12] and a core radius of 390 km, but yielded a crustal thickness of 43 km, close to the value inferred from GRAIL observations. To fit the core radius used in this work, we assume the crustal Th abundance (1488.9 ppb) and the bulk Th abundance (125 ppb) to be constant. Based on mass conservation, the Th abundance of lunar mantle and enrichment factor of the lunar crust are determined as 33.9 ppb and 43.9 respectively. The heat production rate Q is given by

$$Q = C_{Th} \sum_i \left(\frac{C_i}{C_{Th}}\right) \exp\left(-\frac{\ln 2}{t_i} t\right) H_i \tag{32}$$

where C_{Th} is the abundance of Th, C_i is the abundance of the i-th radioactive isotope (i = ^{232}Th, ^{235}U, ^{238}U and ^{40}K), t_i is the half-decaying period of the i-th isotope, H_i is the heat production rate of the i-th radioactive isotope. The values of Th/U and K/U of the bulk Moon are given in Table S1.

In our model, the heat production rate and water content of the lunar mantle are set to their individual volumetrically-averaged values across the whole lunar mantle. This simplification is due to the use of the 1-D parameterised mantle convection model, where the convective mantle is considered as a whole body exchanging heat through its two boundary layers. Hence, radial variations of radioactive element abundances and water content, as well as their variations caused by the mantle flows, are neglected.

Supplementary Text 4. Influence of semi-major axis on lunar thermal evolution

Since the evolution of the lunar orbit is not well constrained, we model the variations of the semi-major axis with an exponential function of time, i.e.

$$a(\tau) = A - (A - a_0) \exp\left(\frac{\tau}{\Gamma}\right), \tag{33}$$

where

$$A = \frac{a_s - a_0 \exp(\tau_s/\Gamma)}{1 - \exp(\tau_s/\Gamma)}, \tag{34}$$

τ is the time in Ga, a_s is the semi-major axis at the end of LMO crystallisation, τ_s is the corresponding time, i.e. $\sim$ 4.2 Ga, a_0 is the present semi-major axis ($\sim 60.2 R_e$), and Γ is a factor used to control the rate of semi-major axis increase.

Table S1: Parameter values of the thermal evolution model.

Parameter	Meaning	Value	Unit
r_p	planetary radius	1740	km
r_c	core radius	352	km
D_{cr}	thickness of anorthosite crust	45	km
ρ_{cr}	crust density	2715	kg/m^3
ρ_m	mantle density	3204	kg/m^3
ρ_c	core density	7200	kg/m^3
c_{pm}	mantle heat capacity	1600	J/(kg·K)
$c_{p,melt}$	melt heat capacity	1600	J/(kg·K)
c_{pc}	core heat capacity	780	J/(kg·K)
L	specific latent heat	6×10^5	J/kg
K_{cr}	crust thermal conductivity	2.0	W/(m·K)
K_m	mantle thermal conductivity	4.0	W/(m·K)
α_m	mantle thermal expansivity	3×10^{-5}	K^{-1}
T_s	surface temperature	250	K
Th/U	ratio between Th abundance and U abundance	3.78	–
K/U	ratio between K abundance and U abundance	3030.30	–
X_w^m	mantle water content	1–100	ppm
D_w	water partitioning coefficient	0.01	–
η_r	mantle reference viscosity	10^{20}–10^{21}	Pa s
E	mantle activation energy	300	kJ/mol
V	mantle activation volume	20×10^{-6}	m^3/mol

By using a reference viscosity of 10^{20} Pa s, a bulk water content of 100 ppm and zero superheating of the initial mantle temperature (i.e. $\Delta T_m = 0$), we perform a parameter study to test the influence of a_s and Γ on the lunar thermal evolution. Figure S2 shows the time evolution of mantle temperature and mantle structure for a case where core-mantle friction is neglected. In this case no partial melting of the upper mantle occurs. We then check the lunar thermal evolution with the core-mantle friction. Figure S3a shows the time evolution of the mantle temperature for different a_s and Γ values. Increasing a_s tends to reduce the mantle temperature, whereas increasing Γ tends to increase it. Figure S3b shows the corresponding variations of the frictional power over time. Figure S4 shows the corresponding evolution of mantle melting. The variation of the two parameters can affect significantly the extent and duration of mantle melting. Increasing a_s tends to reduce the extent and shorten the duration of mantle melting, whereas increasing Γ acts in the opposite direction tends to enlarge the extent and prolong the duration of mantle melting.

Supplementary Text 5. Influences of crustal Th abundance on mantle melting

As an example, we choose the wet-Moon case with $\eta_r = 10^{21}$ Pa s, $a_s = 37R_e$, $\Gamma = 2.0$, and $\Delta T_m = 90$ K to examine the influence of crustal Th abundance on the evolution of mantle melting. Figure S5a shows the evolution of mantle melting for the nominal case, whereas Figure S5b show the evolution of mantle melting considering a crustal Th abundance 10 times greater than that in the nominal case. For the case with higher crustal Th abundance, the maximum melting depth occurs at 3.94 Ga and is 516 km, slightly shallower than the maximum melting

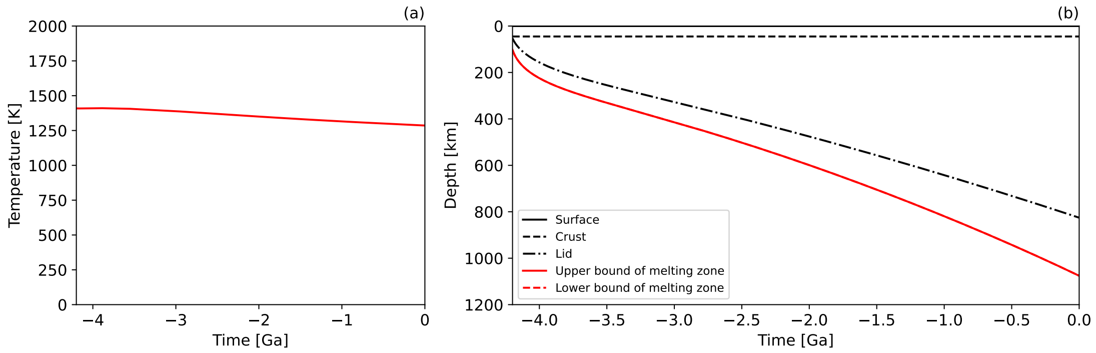

Figure S2: Time evolution of (a) mantle temperature, and (b) mantle structure for a modelling case with a reference viscosity of 10^{20} Pa s, a bulk water content of 100 ppm, zero super-heating in the initial lunar mantle and no core-mantle friction.

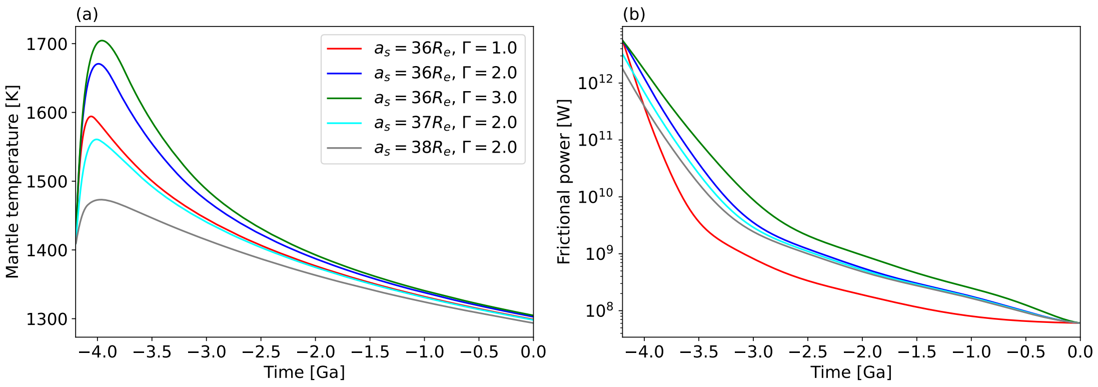

Figure S3: Time evolution of (a) mantle temperature, and (b) frictional power for different values of a_s and Γ. The reference viscosity, bulk water content and super-heating of the initial mantle temperature are 10^{20} Pa s, 100 ppm and zero respectively.

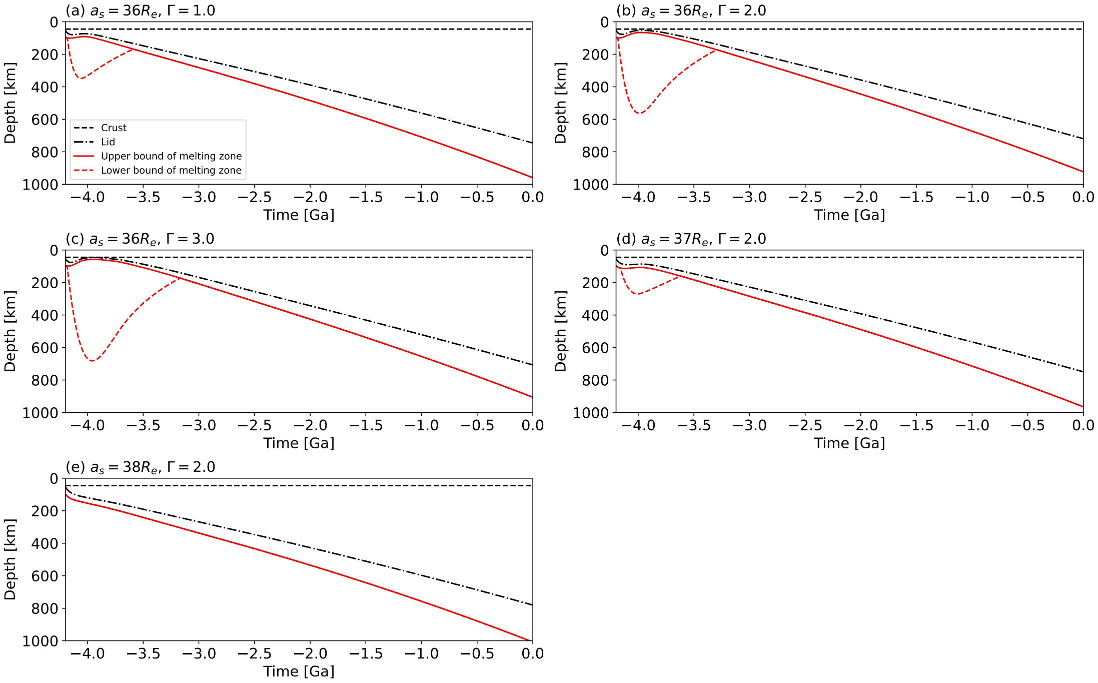

Figure S4: Time evolution of lunar mantle structure for different values of a_s and Γ. Here, mantle reference viscosity, bulk water content and initial increase of mantle temperature are 10^{20} Pa s, 100 ppm and zero respectively. Increasing a_s tends to reduce the extent and shorten the duration of mantle melting, whereas increasing Γ tends to strengthen mantle melting and extend the duration of mantle melting.

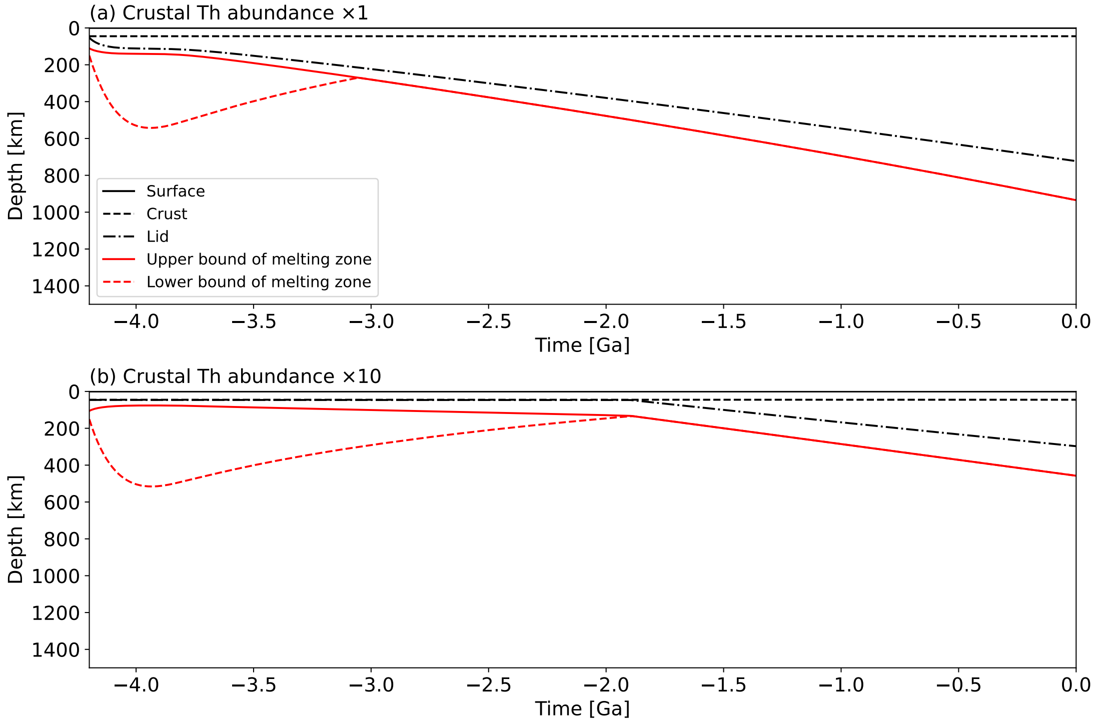

Figure S5: Time evolution of lunar mantle melting for the wet-Moon case with $\eta_r = 10^{21}$ Pa s, $a_s = 37R_e$, $\Gamma = 2.0$ and $\Delta T_m = 90$ K. (a) Nominal case and (b) the case with 10 times larger crustal Th abundance.

depth in the nominal case, i.e. 543 km. Such a shallower maximum melting depth is due to to the thinning of stagnant lid caused by the stronger radiogenic heating in the lunar crust, but the magnitude is nearly negligible with respect to the maximum melting depth.

Supplementary Text 6. Melting near core-mantle friction

In the early phase of lunar evolution, the high power of core-mantle friction can rapidly elevate the CMB temperature. When the CMB temperature reaches the local solidus temperature, the deep lunar mantle begins to melt if frictional heat cannot be dissipated by the heat conduction through the lower boundary layer. Previous works suggested that mantle melting can weaken core-mantle friction [13]. However, core-mantle friction is a special type of precession-driven solid-liquid friction associated with turbulence at the CMB. In the presence of a molten layer, turbulence would no longer occur. However, friction at the interface between molten and solid mantle will begin to take place near the top of the molten layer. Because the kinematic viscosity of magma ($\sim 10^{-3}$–10^{-2} m^2/s) is typically higher than that of liquid iron ($\sim 10^{-6}$ m^2/s), magma-mantle friction would be characterized by a higher frictional coefficient (i.e. κ in Eq. (1) of main text) and therefore might be even stronger than the core-mantle friction (see Eq. 58a–b in [9]).

Whether or not magma-mantle friction can take place depends on the stability of the molten layer, which is determined by the competition between friction-driven melt production and melt

percolation. The velocity of melt percolation can be evaluated by [14]

$$u_{perc} = \frac{\Delta\rho g d^2 \psi}{150\eta_l (1-\psi)}, \tag{35}$$

where $\Delta\rho$ is the solid-liquid density difference, g is the gravitational acceleration, d is the grain size, ψ is the porosity, η_l is the liquid viscosity. Here we set $\eta_l = 1$ Pa s for the melt. Besides, our results suggest a reference viscosity of 10^{20}–10^{21} Pa s for the lunar mantle, which corresponds to a grain size around 10^{-2} m [6]. By a pressure of ~ 4 GPa near the CMB, solid grains should pack very closely and the porosity should approach the value of random close packing (RCP) ~ 0.36 [15]. For $\Delta\rho \sim 10^2$ kg/m^3, the velocity of melt percolation is estimated as $\sim 10^{-5}$ m/s. Correspondingly, the volumetric melt percolation rate is given by $\sim 4\pi r_c^2 u_{perc}$ and thus is $\sim 10^6$ m^3/s. The melt begins to be produced when the CMB temperature reaches the local solidus temperature. By neglecting heat conduction through the lower boundary layer, the melt production rate associated with core-mantle friction can be evaluated by $\sim P_f/\rho_m L$. Given the maximum frictional power in the early phases of lunar evolution ($\sim 10^{13}$ W), the melt production rate is $\sim 10^5$ m^3/s at most. Note that this rate is likely over-estimated due to the neglect of the heat dissipation through the lower boundary layer. As the volumetric rate of percolation is far greater than the melt production rate, the friction-generated melt is not stable at the CMB and can percolate upwards.

Melt percolation has three consequences in the thermal evolution model. First, core-mantle friction can always take place and does not evolve into magma-mantle friction as we referred above. Second, the CMB temperature will not increase further after reaching the local solidus temperature, as Eq. (20) indicates. Third, the percolated melt allows an extra heat transfer from the CMB to the overlying mantle.

The influence of the power due to percolating melt on the thermal evolution of lunar mantle is parameterised as follows. By the energy conservation, the rate of friction-driven melt production can be written as

$$\frac{dV_{melt,c}}{dt} = \frac{P_f - 4\pi r_c^2 q_{mb}}{\rho_m L}. \tag{36}$$

Accordingly, the heat flux associated with melt percolation is

$$q_{perc} = \frac{P_f}{4\pi r_c^2} - q_{mb}. \tag{37}$$

Percolated melt not only transports heat into the lunar mantle, but also tends to increase the total amount of melt, which complicates the mathematical treatment of the problem. Here, we assume that percolated melt freezes at the bottom of the stagnant lid, i.e. at the upper boundary of the convective mantle, and thus account for an extra term an extra in the energy conservation equation, namely P_{perc} in Eq. (1). Accordingly, the power budget P_{perc} can be calculated as

$$P_{perc} = [\rho_m L + \rho_m c_{pm} (T_c - T_l)] \cdot \frac{dV_{melt,c}}{dt}. \tag{38}$$

Supplementary Text 7. Lunar thermal evolution without melt percolation

For comparison, we also evaluate the time evolution of mantle melting neglecting the effect of melt percolation, i.e. with $dV_{perc}/dt = 0$ m^3/s all the time. In all modeling cases, as expected

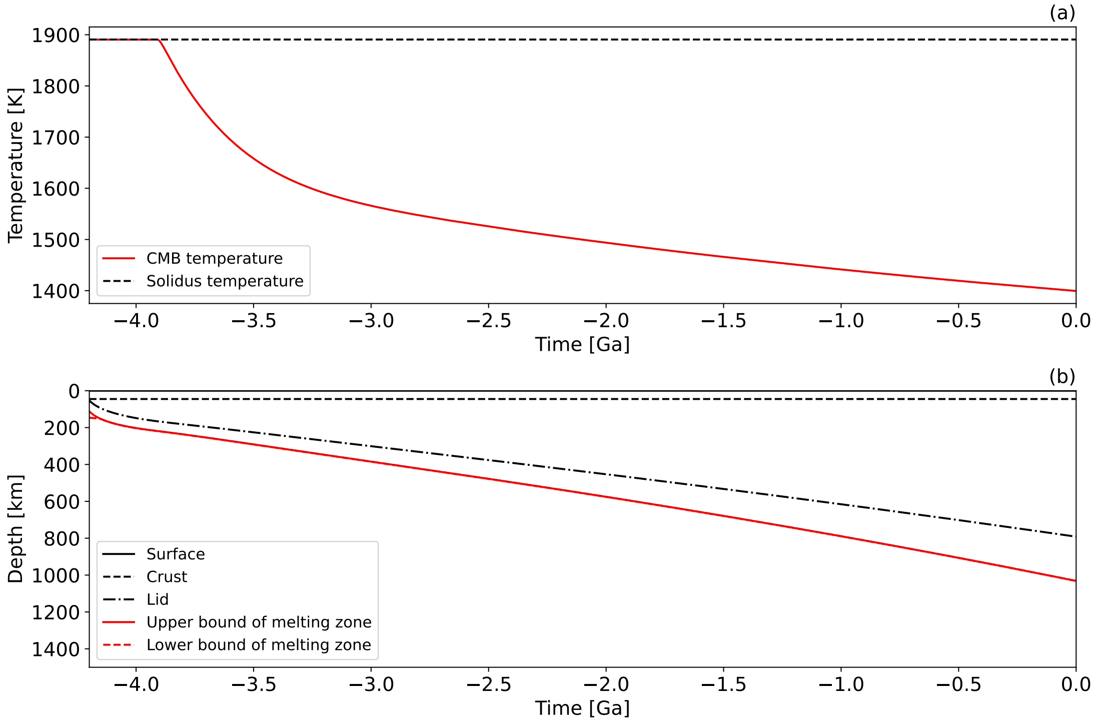

Figure S6: (a) The time evolution of CMB temperature for the percolation-free wet-Moon case with $\eta_r = 10^{21}$ Pa s, $a_s = 37R_e$, $\Gamma = 2.0$ and $\Delta T_m = 90$ K. (b) The time evolution of mantle melting in the same case.

the neglect of melt percolation results in a much shorter duration of global mantle melting owing to a substantial loss of frictional heat. As an example, Figure S6 shows the time evolution of CMB temperature and mantle melting for the wet-Moon case with $\eta_r = 10^{21}$ Pa s, $a_s = 37R_e$, $\Gamma = 2.0$ and $\Delta T_m = 90$ K, as we shown in Figure 3 of the main text. The CMB temperature is cut to the local solidus temperature before 3.91 Ga and decays afterwards. However, in this case mantle melting only lasts until 4.15 Ga instead of 3.06 Ga when melt percolation is included, which emphasises the importance of this factor in maintaining long-lived mantle melting.

According to the discussion of the main text, upper mantle melting should be maintained until $\sim$ 3.0 Ga in order to account for the most intense phase of lunar volcanism [16, 17]. If melt percolation is neglected, the parameter space allowing for long-lived mantle melting is much more restricted being essentially limited to dry cases with a high reference viscosity of $\eta_r = 10^{21}$ Pa s and a high initial mantle temperature of $\Delta T_m = 240$ K (Figure S7).

Supplementary Text 8. Influence of melt on the lunar thermal evolution

In the modelling cases discussed above, we do not consider any rheological weakening caused by the presence of melt. In fact, the parameterized mantle convection approach that we employed

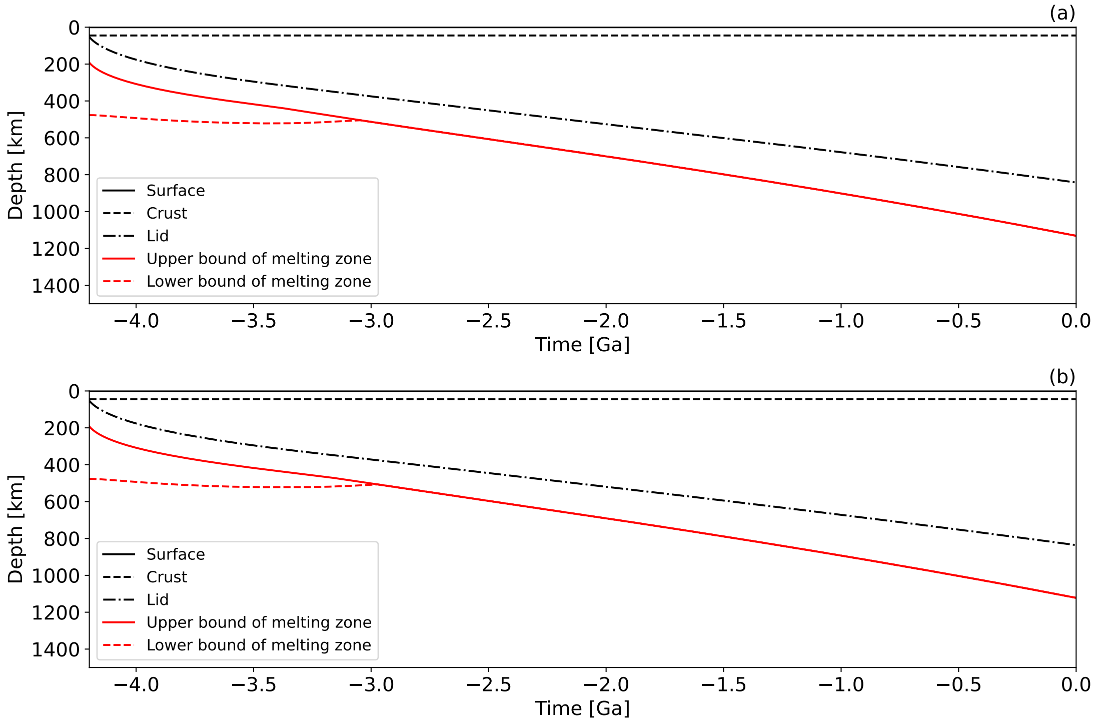

Figure S7: Time evolution of mantle melting for the percolation-free dry-Moon case with $\eta_r = 10^{21}$ Pa s, $a_s = 34R_e$ and $\Delta T_m = 240$ K. The value of Γ is (a) 2.0, and (b) 3.0. Melt percolation from the core-mantle boundary is neglected here.

is designed to deal with a single-phase fluid. The absence of melt dependence of the viscosity is thus an important shortcoming of such a kind of model. As a first-order approximation to treat the effect of melt on the lunar thermal evolution, we reduce the reference viscosity by multiplying it by a melt-fraction-dependent term, i.e.

$$\eta_r' = \eta_r \exp\left(-a\phi_{avg}\right) \tag{39}$$

where $a = 26.0$ [6], and ϕ_{avg} is the melt fraction, volumetrically averaged across the whole convective mantle.

Strictly speaking, we do not find successful cases satisfying all of the constraints listed in the main text. However, two dry-Moon cases with $\eta_r = 10^{21}$ Pa s can still reproduce the constraints from the relaxation of pre-Nectarian basins and the maximum melting depth suggested by the lunar picritic glasses, but show present-day mantle temperatures generally lower than those inferred from the electrical data (Figure S8). Nevertheless, here we still consider these cases as successful. In both cases, mantle melting ceases at ~ 3.3 Ga, generally earlier than in cases that do not include the melt dependence of the viscosity. Figure S9 shows the time evolution of mantle melting for these two cases.

Supplementary Text 9. Influences of tidal dissipation

In our model, we do not consider the influence of tidal dissipation on the lunar thermal evolution. Nevertheless, the contribution of tidal dissipation to the energy budget of the mantle is secondary with respect to that of core-mantle friction and radiogenic heating. Here we use two previous estimates of the power of tidal dissipation by assuming the eccentricity of lunar orbit to be zero and 0.055, i.e. the present value [18]. Recently, it has been suggested that the eccentricity of the lunar orbit was as high as ~ 0.1 in the early phases of the evolution [19]. Hence, we also evaluate the power of tidal dissipation by assuming the eccentricity to be 0.1. The power dissipated within the Moon due to the tides raised by the Earth is evaluated as [20]:

$$P_{tidal} = \frac{21}{2}\frac{k_2}{Q}\frac{GM^2 r_p^5 n}{a^6}\xi(e, I), \tag{40}$$

where $k_2 = 0.024$ [21] and $Q = 100$ [18] are the potential Love number and the effective tidal dissipation parameter of the Moon, G is the gravitational constant, M is the mass of the Earth, n is the mean motion of the lunar orbit, respectively. The parameter $\xi(I, e)$ is a obliquity- and eccentricity-dependent factor, i.e.

$$\xi(I, e) = \frac{2}{7}\frac{f_0(e)}{\beta^{15}} - \frac{4}{7}\frac{f_1(e)}{\beta^{12}}\cos I + \frac{1}{7}\frac{f_2(e)}{\beta^9}(1+\cos^2 I) + \frac{3}{14}\frac{e^2 f_3(e)}{\beta^{13}}\sin^2 I\cos(2\Lambda), \tag{41}$$

where $\beta = \sqrt{1-e^2}$ and

$$\begin{cases} f_0(e) = & 1 + \frac{31}{2}e^2 + \frac{255}{8}e^4 + \frac{185}{16}e^6 + \frac{25}{64}e^8 \\ f_1(e) = & 1 + \frac{15}{2}e^2 + \frac{45}{8}e^4 + \frac{5}{16}e^6 \\ f_2(e) = & 1 + 3e^2 + \frac{3}{8}e^4 \\ f_3(e) = & 1 - \frac{11}{6}e^2 + \frac{2}{3}e^4 + \frac{1}{6}e^6 \end{cases} . \tag{42}$$

Here Λ is a measure of the longitude of the node of the equator on the orbit plane with respect to the pericenter of the orbit. The power of radiogenic heating throughout the entire lunar silicate portion is estimated by

$$P_{radio} = 4\pi\int_{r_c}^{r_p}\rho\left(r\right)Q\left(r\right)r^2 dr. \tag{43}$$

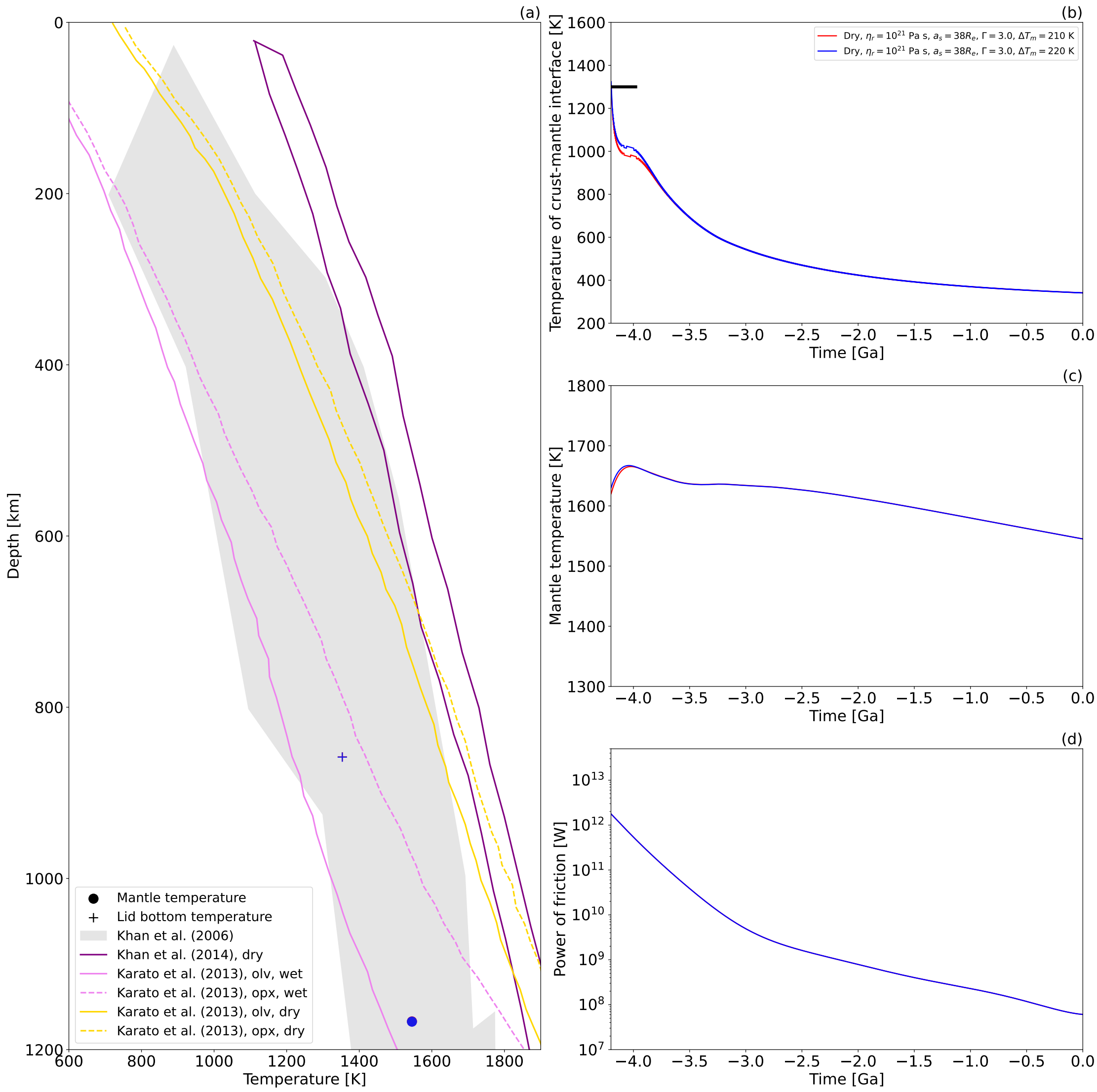

Figure S8: (a) The present-day mantle temperature and lid bottom temperature, (b) the time evolution of temperature at the crust-mantle interface, (c) the time evolution of mantle temperature, and (d) the time evolution of frictional power in the successful cases obtained by the thermal evolution model coupling the viscosity-melt dependence.

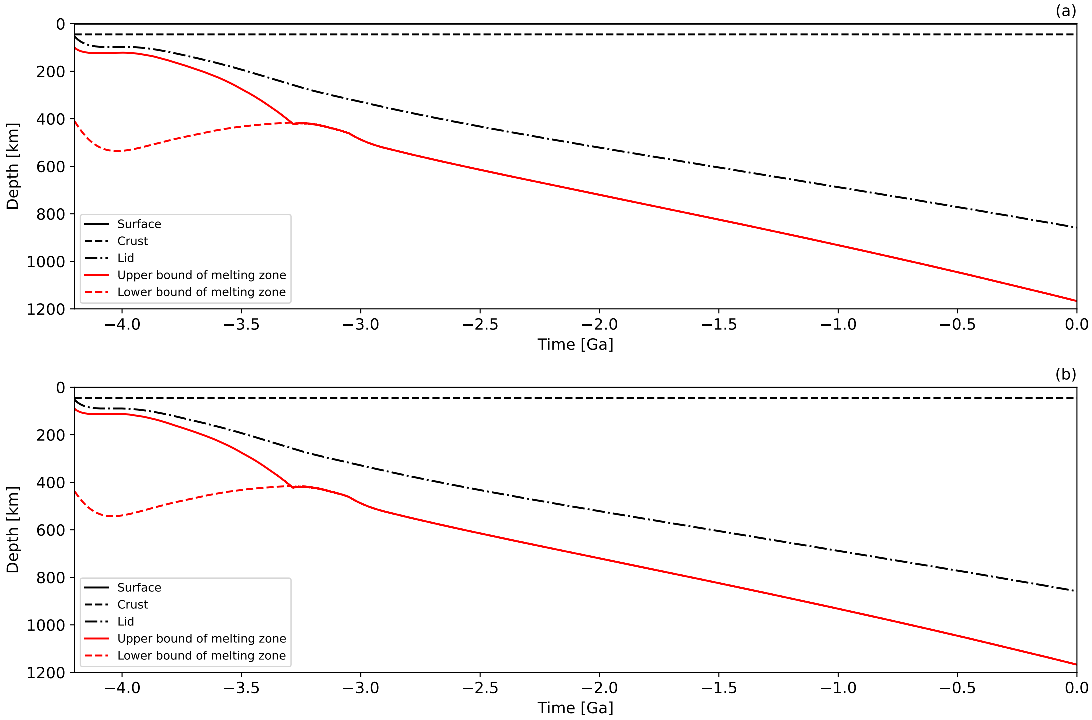

Figure S9: The time evolution of mantle melting for the dry-Moon cases with (a) $\eta_r = 10^{21}$ Pa s, $a_s = 38R_e$, $\Gamma = 3$ and $\Delta T_m = 210$ K, and (b) $\eta_r = 10^{21}$ Pa s, $a_s = 38R_e$, $\Gamma = 3$ and $\Delta T_m = 220$ K.

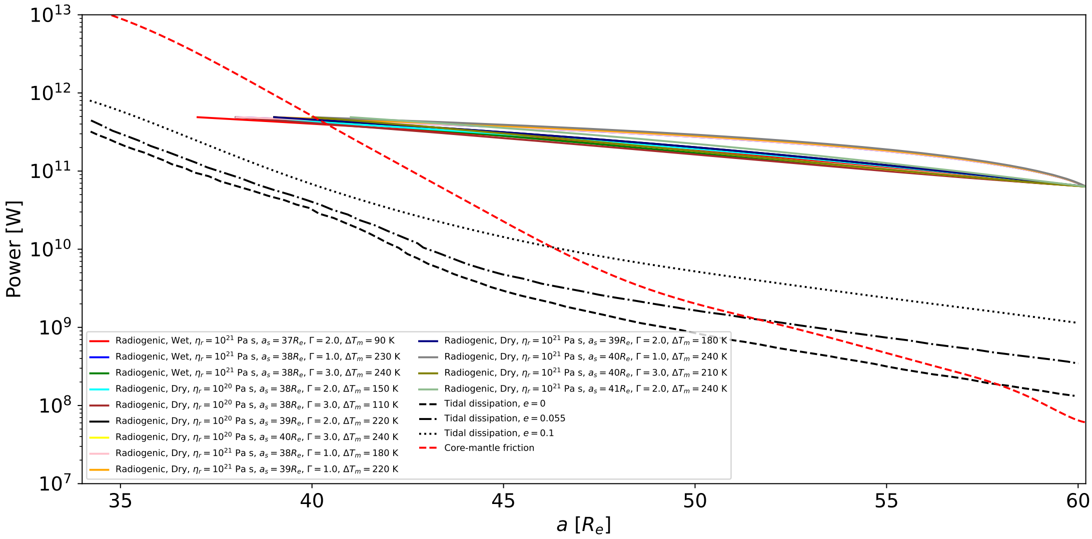

Figure S10: Comparisons between the power of radiogenic heat (coloured solid curves), tidal dissipation (black dashed curves), and core-mantle friction (red dashed curve) for all successful cases. All power budgets are plotted as the functions of the semi-major axis of lunar orbit.

Figure S10 shows the power of radiogenic heat, the power of tidal dissipation and the power of core-mantle friction as the functions of the semi-major axis. As seen from this figure, the power of core-mantle friction is significantly higher than the power of tidal dissipation for $a \lesssim 46R_e$ at least. Until the power of tidal dissipation is greater than the power of core-mantle friction, radiogenic heating dominates the thermal evolution of the Moon. We also note that our thermal evolution modelling begins from 4.2 Ga, i.e. the end of LMO phase. Accordingly, the power of radiogenic heating is also calculated from 4.2 Ga, during which a>37–39R_e for the successful cases. The power of radiogenic heating for a<37–39R_e is non-zero and can also be estimated, but is beyond the scope of this study.

References

[1] N. Tosi, M. Godolt, B. Stracke, T. Ruedas, J. L. Grenfell, D. Höning, A. Nikolaou, A.-C. Plesa, D. Breuer, and T. Spohn, "The habitability of a stagnant-lid earth," *Astronomy & Astrophysics*, vol. 605, p. A71, 2017.

[2] O. Grasset and E. Parmentier, "Thermal convection in a volumetrically heated, infinite prandtl number fluid with strongly temperature-dependent viscosity: Implications for planetary thermal evolution," *Journal of Geophysical Research: Solid Earth*, vol. 103, no. B8, pp. 18171–18181, 1998.

[3] C. Reese, V. Solomatov, and J. Baumgardner, "Scaling laws for time-dependent stagnant lid convection in a spherical shell," *Physics of the Earth and Planetary Interiors*, vol. 149, no. 3-4, pp. 361–370, 2005.

[4] G. Schubert, D. L. Turcotte, and P. Olson, *Mantle convection in the Earth and planets*. Cambridge University Press, 2001.

[5] F. Deschamps and C. Sotin, "Inversion of two-dimensional numerical convection experiments for a fluid with a strongly temperature-dependent viscosity," *Geophysical Journal International*, vol. 143, no. 1, pp. 204–218, 2000.

[6] G. Hirth and D. Kohlstedf, "Rheology of the upper mantle and the mantle wedge: A view from the experimentalists," *Geophysical monograph-american geophysical union*, vol. 138, pp. 83–106, 2003.

[7] R. F. Katz, M. Spiegelman, and C. H. Langmuir, "A new parameterization of hydrous mantle melting," *Geochemistry, Geophysics, Geosystems*, vol. 4, no. 9, 2003.

[8] E. Takahashi, "Speculations on the archean mantle: missing link between komatiite and depleted garnet peridotite," *Journal of Geophysical Research: Solid Earth*, vol. 95, no. B10, pp. 15941–15954, 1990.

[9] J. G. Williams, D. H. Boggs, C. F. Yoder, J. T. Ratcliff, and J. O. Dickey, "Lunar rotational dissipation in solid body and molten core," *Journal of Geophysical Research: Planets*, vol. 106, no. E11, pp. 27933–27968, 2001.

[10] M. A. Wieczorek, G. A. Neumann, F. Nimmo, W. S. Kiefer, G. J. Taylor, H. J. Melosh, R. J. Phillips, S. C. Solomon, J. C. Andrews-Hanna, S. W. Asmar, *et al.*, "The crust of the moon as seen by grail," *Science*, vol. 339, no. 6120, pp. 671–675, 2013.

[11] S. Yu, N. Tosi, S. Schwinger, M. Maurice, D. Breuer, and L. Xiao, "Overturn of ilmenite-bearing cumulates in a rheologically weak lunar mantle," *Journal of Geophysical Research: Planets*, vol. 124, no. 2, pp. 418–436, 2019.

[12] H. S. C. O'neill, "The origin of the moon and the early history of the earth—a chemical model. part 1: The moon," *Geochimica et Cosmochimica Acta*, vol. 55, no. 4, pp. 1135–1157, 1991.

[13] C. Dwyer, D. Stevenson, and F. Nimmo, "A long-lived lunar dynamo driven by continuous mechanical stirring," *Nature*, vol. 479, no. 7372, pp. 212–214, 2011.

[14] V. Solomatov, "Magma oceans and primordial mantle differentiation," *Evolution of the Earth*, vol. 9, pp. 91–119, 2007.

[15] R. Schräpler, J. Blum, I. von Borstel, and C. Güttler, "The stratification of regolith on celestial objects," *Icarus*, vol. 257, pp. 33–46, 2015.

[16] H. Hiesinger, J. Head III, U. Wolf, R. Jaumann, and G. Neukum, "Ages and stratigraphy of mare basalts in oceanus procellarum, mare nubium, mare cognitum, and mare insularum," *Journal of Geophysical Research: Planets*, vol. 108, no. E7, 2003.

[17] S. Kato, T. Morota, Y. Yamaguchi, S. I. Watanabe, H. Otake, and M. Ohtake, "Magma source transition of lunar mare volcanism at 2.3ga," *Meteoritics and Planetary Science*, 2017.

[18] S. Peale and P. Cassen, "Contribution of tidal dissipation to lunar thermal history," *Icarus*, vol. 36, no. 2, pp. 245–269, 1978.

[19] H. Daher, B. K. Arbic, J. G. Williams, J. K. Ansong, D. H. Boggs, M. Müller, M. Schindelegger, J. Austermann, B. D. Cornuelle, E. B. Crawford, *et al.*, "Long-term earth-moon evolution with high-level orbit and ocean tide models," *Journal of Geophysical Research: Planets*, vol. 126, no. 12, p. e2021JE006875, 2021.

[20] J. Wisdom, “Tidal dissipation at arbitrary eccentricity and obliquity,” *Icarus*, vol. 193, no. 2, pp. 637–640, 2008.

[21] J. G. Williams, A. S. Konopliv, D. H. Boggs, R. S. Park, D.-N. Yuan, F. G. Lemoine, S. Goossens, E. Mazarico, F. Nimmo, R. C. Weber, S. W. Asmar, H. J. Melosh, G. A. Neumann, R. J. Phillips, D. E. Smith, S. C. Solomon, M. M. Watkins, M. A. Wieczorek, J. C. Andrews-Hanna, J. W. Head, W. S. Kiefer, I. Matsuyama, P. J. McGovern, G. J. Taylor, and M. T. Zuber, “Lunar interior properties from the grail mission,” *Journal of Geophysical Research: Planets*, vol. 119, no. 7, pp. 1546–1578, 2014.
